# Schisandrol A Suppresses Catabolic Factor Expression by Blocking NF-κB Signaling in Osteoarthritis

**DOI:** 10.3390/ph14030241

**Published:** 2021-03-08

**Authors:** Seong Jae Han, Jimoon Jun, Seong-il Eyun, Choong-Gu Lee, Jimin Jeon, Cheol-Ho Pan

**Affiliations:** 1 Department of Biomedical Sciences, Graduate School of Medicine, Ajou University, Suwon 16499, Korea; hsj2018@ajou.ac.kr; 2 Department of Pharmacology, School of Medicine, Ajou University, Suwon 16499, Korea; 3 Degenerative InterDiseases Research Center, School of Medicine, Ajou University, Suwon 16499, Korea; 4 Department of Life Science, Chung-Ang University, Seoul 06974, Korea; zmfozld123@cau.ac.kr; 5 Natural Product Informatics Research Center, Korea Institute of Science and Technology, Gangneung 25451, Korea; 6 Division of Bio-Medical Science & Technology, KIST School, Korea University of Science and Technology (UST), Gangneung 02792, Korea

**Keywords:** Schisandrol A, cartilage destruction, osteoarthritis, NF-κB

## Abstract

Schisandrol A possesses pharmacological properties and is used to treat various diseases; however, its effects on osteoarthritis (OA) progression remain unclear. Here, we investigated Schisandrol A as a potential therapeutic agent for OA. In vitro, Schisandrol A effects were confirmed based on the levels of expression of catabolic factors (MMPs, ADAMTS5, and Cox2) induced by IL-1β or Schisandrol A treatment in chondrocytes. In vivo, experimental OA in mice was induced using a destabilized medial meniscus (DMM) surgical model or oral gavage of Schisandrol A in a dose-dependent manner, and demonstrated using histological analysis. In vitro and in vivo analyses demonstrated that Schisandrol A inhibition attenuated osteoarthritic cartilage destruction via the regulation of Mmp3, Mmp13, Adamts5, and Cox2 expression. In the NF-κB signaling pathway, Schisandrol A suppressed the degradation of IκB and the phosphorylation of p65 induced by IL-1β. Overall, and Schisandrol A reduced the expression of catabolic factors by blocking NF-κB signaling and prevented cartilage destruction. Therefore, Schisandrol A attenuated OA progression, and can be used to develop novel OA drug therapies.

## 1. Introduction

Joint inflammation and cartilage destruction are the leading causes of osteoarthritis (OA) and are responsible for making a patient’s daily life uncomfortable [1]. OA disease includes features such as cartilage destruction, subchondral bone sclerosis, synovial inflammation, meniscal degeneration, and fibrosis of the infrapatellar fat pad [2,3]. In addition, OA has several risk factors such as aging, gender, obesity, and reactive oxygen species (ROS) production, which influence a variety of processes and lead to joint destruction. Such factors increase susceptibility to cell death leading to defect repair of the damaged matrix and inflammatory and catabolic conditions, in turn, promoting the development of OA by imbalance in joints [4,5,6]. OA is a degenerative disorder of cartilage that is primarily caused by the collapse of cartilage homeostasis [7]. In articular cartilage cells, the molecular mechanisms of inflammation and cartilage destruction are driven by the induction of catabolic factors such as matrix metalloproteinases (MMPs), aggrecanase, and cyclooxygenase 2 (COX-2) [8]. Pro-inflammatory cytokines (e.g., interleukin-1β (IL-1β)) and mechanical stress can induce MMPs, ADAMTS, and COX-2 expression and thereby, promote cartilage destruction and OA development [9,10].

There are many MMP isotypes, among which MMP3 and MMP13 are crucial molecules that degrade the cartilage matrix. MMP3 and MMP13 both have aggrecanase and collagenase activities [11,12] and are responsible for the degradation of aggrecan, type II collagen, and other extracellular matrix (ECM) components. [11] Aggrecanases are members of the A disintegrin and metalloproteinase with thrombos-pondin motifs (ADAMTS) family. ADAMTS are responsible for aggrecan degradation in OA and ADAMTS5 is a particularly important aggrecanase [13]. COX-2 is primarily associated with joint inflammation, which activates MMP3 and MMP13 and eventually accelerates ECM destruction during OA development [14,15]. Therefore, as the levels of COX-2, MMP3, and MMP13 increase during OA progression, the ECM decreases, leading to the onset of severe OA and its resultant symptoms.

IL-1β regulates the catabolism of chondrocytes by activating nuclear factor (NF)-κB signaling, which later leads to OA [16,17]. As a transcription factor, NF-κB is primarily responsible for regulating biological processes such as inflammation, cell differentiation, and proliferation [18,19]. The results of many prior studies revealed that activation of the NF-κB signaling pathway increases the expression of MMP3, MMP13, and COX-2 which are known to be involved in the development of OA through the downstream induction of catabolic factors in chondrocytes [19,20].

*Schisandra chinensis* Baill. is a traditional Chinese medicine that has been recorded as a drug and is used to treat various diseases [21,22]. The lignan Schisandrol A, an active ingredient derived from the fruit of *S. chinensis* Baill. [23,24,25,26] has been widely reported to possess a variety of pharmacological attributes, including antioxidant, anti-apoptotic, and anti-allergic properties [27,28,29]. In addition, results of a previous study revealed that Schisandrol A exhibited anti-inflammatory effects that involved pro-inflammatory mediators (e.g., COX-2) via inhibition of NF-κB activation [30]. There is evidence that Schisandrol A can ameliorate symptoms of breast cancer, Alzheimer’s disease, and liver disease [27]. In other studies, Schisandrol A was shown to exert protective effects against hypoxia/reoxygenation-induced vascular endothelial damage and glutamate-induced excitotoxicity [31,32]. Although Schisandrol A is used to treat a variety of disorders, it has not been reported to be effective in the treatment of OA, and its role as an OA therapeutic agent is currently unknown.

Currently available treatments for OA include steroidal anti-inflammatory drugs (NSAIDs), which exhibit side effects and toxicity [6]. Consequently, novel OA treatments with little or no side effects are required. The purpose of the present study was to assess the capacity of Schisandrol A to attenuate MMP3, MMP13, and COX-2 expression via NF-κB signaling and, in turn, inhibit OA development, which was accomplished through in vitro and in vivo experiments. No previous studies have explored the potential effects of Schisandrol A on OA. Therefore, we investigated the potential use of Schisandrol A in the development of novel drugs for degenerative OA.

## 2. Results

### 2.1. Schisandrol A Is Not Toxic to Chondrocytes

**Figure 1:**
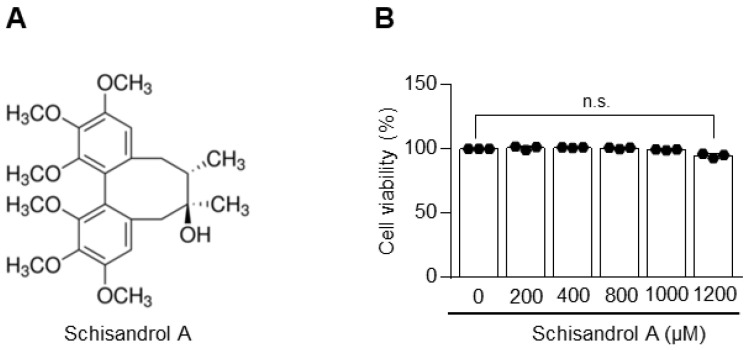
Toxicity of Schisandrol A toward chondrocytes. (**A**) Chemical structure of Schisandrol A. (**B**) Toxic effects of Schisandrol A on chondrocytes. Cell viability was measured at different concentrations for 24 h and analyzed using a lactate dehydrogenase (LDH) assay. Data were analyzed using one-way ANOVA with Bonferroni’s test, and plotted values were expressed as means ± SEM; n.s. *p* > 0.05, compared to the control group.

First, we examined whether Schisandrol A is toxic to chondrocytes. There was little difference in cell viability between the groups treated with Schisandrol A 200, 400, 800, 1000, and 1200 μM) and the control group, with the exception of a small but noticeable reduction at 1200 μM (Figure 1). Based on these data, all subsequent experiments were conducted using concentrations of 400, 800, and 1000 μg/mL.

### 2.2. Schisandrol A Inhibits IL-1β-Induced MMPs and COX-2 in Chondrocytes

**Figure 2:**
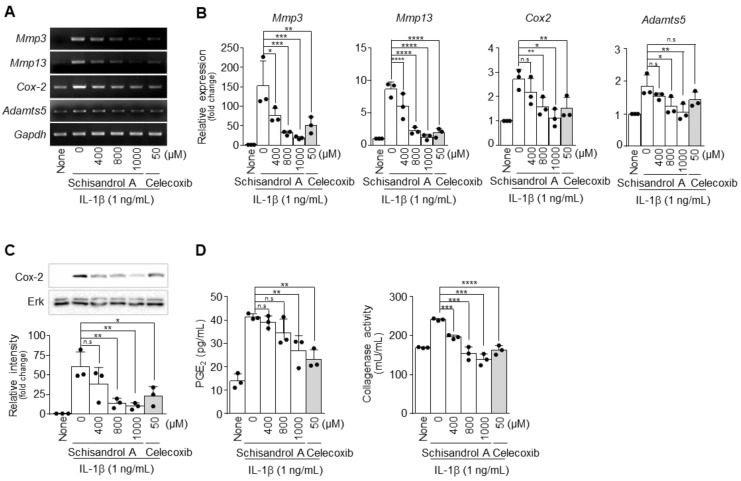
Schisandrol A suppresses expression of matrix metalloproteinases (MMPs) and cyclooxygenase 2 (COX-2) and decreases IL-1β-induced production of PGE_2_ and collagenase in mouse articular chondrocytes. Chondrocytes stimulated with IL-1β (1 ng/mL) were treated with or without various concentrations of Schisandrol A (400, 800, or 1000 µM). The mRNA expression levels of *MMP3*, *MMP13*, *COX-2* and *ADAMTS5* were determined by RT-PCR (**A**) and qRT-PCR (**B**). The protein expression of COX-2 was measured by Western blot and densitometry (**C**). Celecoxib (50 µM) was used as a positive control. PGE_2_ (**D**, left panel) and collagenase (**D**, right panel) assays were performed on chondrocytes stimulated with IL-1β (1 ng/mL) and treated with or without Schisandrol A at various concentrations (400, 800, or 1000 µM). Celecoxib (50 µM) was used as a positive control. Glyceraldehyde 3-phosphate dehydrogenase (*GAPDH*) and extracellular signal-regulated kinase (ERK) were used as loading controls. Data were analyzed using one-way ANOVA with Bonferroni’s test, and plotted values were expressed as means ± SEM; n.s. *p* > 0.05, * *p* < 0.05, ** *p* < 0.01, *** *p* < 0.001 and **** *p* < 0.0001 compared to the control group.

The levels of MMP3, MMP13, COX-2, and ADAMTS5 increased sharply in chondrocytes after IL-1β treatment [19,20]. However, when the chondrocytes were co-treated with Schisandrol A for 24 h, the mRNA levels of *MMP3*, *MMP13*, *COX-2,* and *ADAMTS5* gradually decreased in a concentration-dependent manner, as determined by RT-PCR (Figure 2A) and qRT-PCR (Figure 2B). PCR primers are summarized in Table S1. In addition, the protein expression levels of COX-2 decreased significantly at 800 and 1000 μM of Schisandrol A, as determined by Western blot (Figure 2C). Chondrocytes were treated with IL-1β in the absence or presence of different concentrations of Schisandrol A, followed by prostaglandin E2 (PGE_2_) and collagenase assays. The assay results showed that synthesis of PGE_2_ and collagenase was dramatically decreased by Schisandrol A in a concentration-dependent manner (Figure 2D). Considering the results above, treatment with Schisandrol A reduced the expression of various catabolic factors compared to IL-1β treatment and suggest that Schisandrol A has the potential to alleviate OA treatment.

### 2.3. Oral Gavage of Schisandrol A Inhibits Cartilage Degradation in the Destabilization of the Medial Meniscus (DMM)-Induced Arthritis Model

**Figure 3:**
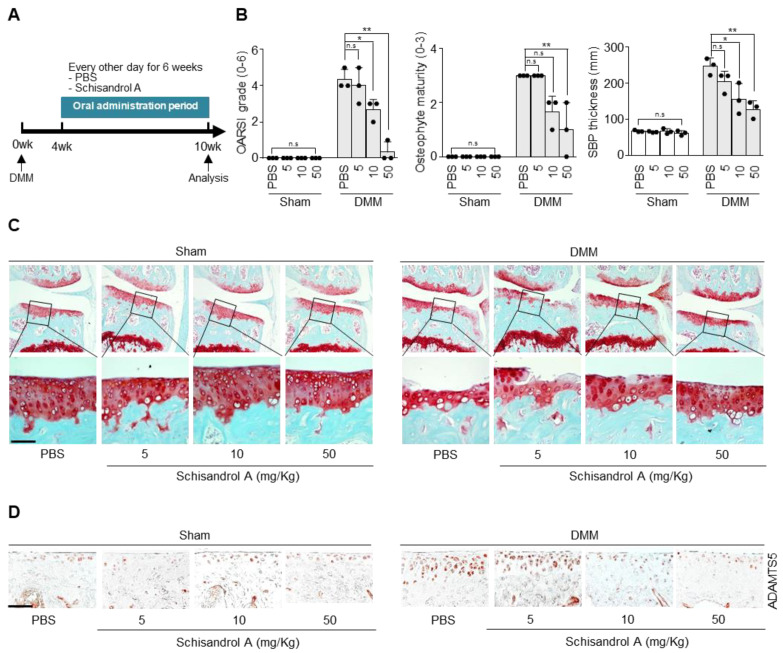
Oral administration of Schisandrol A protects against cartilage degradation in osteoarthritis (OA). (**A**) Overall experimental plan for the analysis of the DMM-induced OA model (*n* = 3 for each group). Mice were administered PBS or Schisandrol A (5, 10, or 50 mg/kg) every other day from 4 weeks after DMM surgery until they were analyzed at 10 weeks. (**B**) Cartilage degradation was determined by Osteoarthritis Research Association International (OARSI) score, osteophyte maturity, and subchondral bone plate thickness (SBP) at 10 weeks after DMM surgery. Data were analyzed using one-way ANOVA with Bonferroni’s test, and plotted values were expressed as means ± SEM; n.s. *p* > 0.05, * *p* < 0.05 and ** *p* < 0.01 compared to the control (PBS) group. (**C**) Analysis of cartilage degradation was performed by safranin O staining. (**D**) Expression of ADAMTS5 in cartilage of DMM-induced OA model is determined with immunostaining. Scale bar = 100 μm.

To determine whether oral administration of Schisandrol A prevents arthritic cartilage destruction in vivo, we investigated the effect of the compound in the DMM-induced arthritis mouse model. The different treatments are shown in Figure 3A. Osteoarthritis Research Association International (OARSI) grade, osteophyte maturity, and subchondral bone plate thickness were significantly lower in the Schisandrol A-treated groups than in the control group, except for the group treated with 5 mg/kg Schisandrol A. Moreover, compared with the PBS control, oral gavage of Schisandrol A noticeably prevented cartilage destruction (Figure 3C) and decreased ADAMTS5 expression based on immunohistochemical staining results (Figure 3D).

### 2.4. Schisandrol A Prevents Activation of IL-1β-Induced NF-κB Signaling in Mouse Articular Chondrocytes

**Figure 4:**
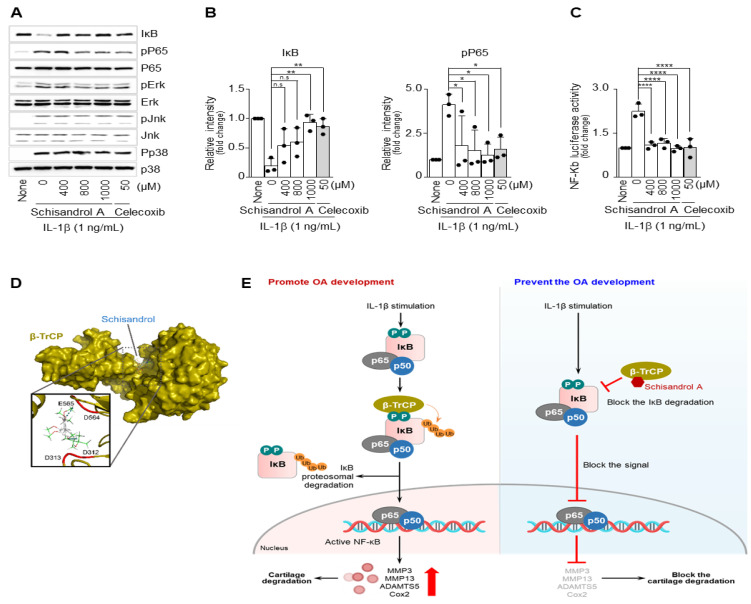
Schisandrol A regulates IL-1β-induced NF-κB activation. Mouse articular chondrocytes were pretreated with Schisandrol A at different concentrations for 24 h (*n* = 3) before being treated with IL-1β (1 ng/mL) for 15 min. (**A**) Protein levels of IκB and phosphorylated p65 (pp65), phosphorylated JNK (pJNK), phosphorylated ERK (pERK) and phosphorylated p38 (pp38) were evaluated by Western blotting. (**B**) Protein levels of IκB and phosphorylated p65 (pp65) were evaluated using densitometry. Extracellular signal-regulated kinase (ERK) and p65 were used as loading controls. NF-κB transcriptional activity (**C**) was assessed using a luciferase reporter gene assay. (**D**) Interaction simulation of Schisandrol A and β-TrCP generated by Autodock Vina program. (**E**) General summary of our findings. Celecoxib (50 µM) was used as a positive control. Data were analyzed using one-way ANOVA with Bonferroni’s test, and the plotted values were expressed as means ± SEM; n.s. *p* > 0.05, * *p* < 0.05, ** *p* < 0.01 and **** *p* < 0.0001 compared to the control group.

We examined whether Schisandrol A regulates the intermediates of the NF-κB signaling pathway. Western blot analysis (Figure 4A) and densitometry measurements (Figure 4B) revealed that Schisandrol A suppressed the degradation of IκB, a factor that regulates NF-κB nuclear translocation, and blocked the phosphorylation of p65, one of the subunits the NF-κB complex. In addition, MAPK signaling is important and is involved in apoptosis of chondrocytes and the degradation of extracellular matrix in OA progression [33]. To evaluate MAPK signaling pathways influenced by Schisandrol A, the expression of p-ERK1/2, p-JNK, and p-p38 proteins were analyzed by Western blotting. However, Schisandrol A did not influence the phosphorylation of ERK1/2, JNK, or p38. We also investigated whether IL-1β-induced activation of NF-κB was attenuated by Schisandrol A. The IL-1β-dependent increase in NF-κB reporter activity was reduced by Schisandrol A co-treatment (Figure 4C). To determine how Schisandrol A regulates catabolic factor by inhibiting NF-κB signaling, we examined the interaction of Schisandrol A with proteins known to regulate NF-κB signaling. The presence of the interaction between Schisandrol A and β-TrCP was confirmed by modeling in silico protein structural homology (Figure 4D and the expected binding site of Shcisandrol A in the β-TrCP amino acid sequence is shown in blue (Supplementary Figure S1). Thus, NF-κB-dependent transcriptional activity was suppressed by Schisandrol A, which would lead to downregulation of MMP3, MMP13, and COX-2 expression in chondrocytes.

## 3. Discussion

OA is one of several diseases caused by exposure to multiple factors and is associated with multiple risks, such as age, joint trauma, and mechanical stress [34,35]. Nonsteroidal anti-inflammatory drugs were first prescribed for the treatment of OA. However, these drugs can cause side effects, such as peptic ulcers, intestinal bleeding, and myocardial infarction [36]. Therefore, it is important to develop safer drugs to treat OA.

Currently, there is no complete cure for OA, and most of the treatments being applied are aimed at controlling pain or maintaining joint function. Numerous studies have reported the protective pharmacological effects of certain agents during joint inflammation, such as carnosine and hyaluronic acid (HA), and anti-oxidative and anti-inflammatory properties, such as *Anacardium occidentale* L., research is ongoing to investigate their potential efficacy [37,38]. Research is also underway to improve OA symptoms by focusing on agents with anti-inflammatory and pain relief properties such as ALIAmide or a combination of hyaluronic acid and adelmidrol [39,40]. Since available pharmaceuticals are limited in terms of efficacy and long-term safety, natural products, which are generally considered safe, have increasingly attracted the interest of stakeholders and researchers.

*S. chinensis* Baill. is a medicinal herb that has been frequently used for the treatment of various diseases [21,22]. The lignan Schisandrol A is one of the active ingredients extracted from this herb [19,20,21,22]. Schisandrol A has been widely reported to possess a variety of pharmacological properties [27,28,29]. It has been previously shown to protect against acute myocardial ischemia through the phosphoinositide 3-kinase (PI3K)/Akt-NADPH oxidase 2 (NOX2) signaling pathway [28]. In addition, Schisandrol A has been used to treat neurological disorders in many studies [41,42,43]. It has been reported to improve learning and memory impairment and attenuate β-amyloid deposition in a mouse model of Alzheimer’s disease by ameliorating neurotransmitter dysfunction [44]. Schisandrol A utilizes an interesting mechanism to protect against ischemia/reperfusion-induced nerve damage by inactivating autophagy that occurs through the 5′-adenosine monophosphate-activated protein kinase-mammalian target of rapamycin (AMPK-mTOR) pathway, and thus can be used as a neuroprotective agent against ischemic stroke [45]. Schisandrol A also attenuates lung injury disease by modulating the Toll-like receptor 4 (TLR-4) and Akt/Forkhead box protein O1 (FoxO1) signaling pathways [46]. Schisandrol A is known to relieve inflammation induced by avian pathogenic *Escherichia coli* in chicken type II pneumonia [47]. In addition, it suppresses the levels of COX-2 and ROS through inhibition of the NF-κB signaling pathway in liver inflammation [48].

There are molecules with structures similar to that of Schisandrol A, mainly schisandrin A and schisandrin B, with Schisandrol A possessing a different hydroxyl group from the other two molecules. In addition, schisandrin A has three methoxy groups on one benzene ring, while schisandrin B has a structure with two methoxy groups connected to form a ring [21]. Similar to Schisandrol A, schisandrin A, and schisandrin B have been evaluated for the treatment of many diseases [49,50,51]. Schisandrin A inhibits the development of OA through inhibition of mitogen-activated protein kinase (MAPK) and NF-κB signaling pathways [52] and is known to exhibit potent anticancer activity in colon cancer cells [53]. Schisandrin B also alleviates OA through inhibition of NF-κB and MAPK signaling pathways [54] and suppresses traumatic spinal cord injury by inhibiting p53 signaling to attenuate the inflammatory response, oxidative stress, and apoptosis caused by such injury [55]. Thus, several molecules with structures similar to that of Schisandrol A have been reported to alleviate OA symptoms [52,53], but the association between Schisandrol A and OA has not been investigated. Therefore, we performed several experiments and found a positive effect of Schisandrol A on OA.

In clinical and experimental OA, cartilage degradation and inflammation are caused by increased expression of catabolic factors, such as MMPs and COX-2 [14,15,16]. The regulators of MMP and COX-2 expression are known as pro-inflammatory cytokines (e.g., IL-1β) [20]. IL-1β has been reported to regulate the expression of catabolic factors in mouse chondrocytes [11,56]. It regulates downstream molecules by activating the NF-κB signaling pathway in chondrocytes and plays an important role in OA progression [57,58]. Therefore, mouse chondrocytes were treated with IL-1β to simulate experimental OA conditions in vitro. Prior to the experiment, we investigated cytotoxicity at various Schisandrol A concentrations. Cytotoxicity was not observed up to 1000 μM (1 mM) in in vitro analysis. Treatment of mouse chondrocytes with IL-1β is known to upregulate the expression of catabolic factors, including MMP3, MMP13, and COX-2 [59]. MMP3 and MMP13 act as collagenases and aggrecanases, promoting the breakdown of type II collagen and degrading cartilage [11]. Prostaglandin synthesis mediated by COX-2 expression is a major risk factor associated with OA, and elevated prostaglandins increase MMP synthesis [59]. According to our current study. Schisandrol A suppressed IL-1β-induced OA progression by reducing the expression and activity of MMP3, MMP13, and COX-2 in chondrocytes. Therefore, Schisandrol A is a feasible therapeutic agent for OA. Our results suggest that Schisandrol A blocks cartilage destruction via inhibition of the NF-κB signaling pathway by inhibiting IκB degradation and p65 phosphorylation, resulting in reduced NF-κB activity.

NF-κB is a transcription factor found in all animal cell types, including chondrocytes [16,60]. NF-κB plays an incredibly important role in the cell’s response to several stimuli including stress, chemokines, and pro-inflammatory cytokines [56,61]. NF-κB-mediated responses are initiated by the degradation of the IκB protein inhibitor bound to NF-κB. After IκB is degraded, the NF-κB complex is phosphorylated and translocated to the nucleus. Then, various mRNAs including *MMP3*, *MMP13*, and *COX-2* are upregulated [60,62,63]. Activation of the NF-κB signaling pathway leads to the degradation of articular cartilage and increases the expression of catabolic factors that can lead to arthritis [64]. Therefore, blocking the NF-κB signaling pathway is considered one way to treat OA. Previous studies have also shown that suppressing the NF-κB signaling pathway reduces the expression of Mmps and Cox-2 [59,65]. According to our experimental results, Schisandrol A inhibited the degradation of IκB and phosphorylation of p65. In order to regulate NF-κB signaling, two major signaling steps are required: Activation of IKK and degradation of phosphorylated inhibitors. IKK activation and IkB degradation involve different ubiquitination modes. β-TrCP induces ubiquitination of IκB, thereby degrading IκB, and NF-κB, which is separated from the IκB-NF-κB complex, enters the nucleus, and NF-κB signaling is activated [66,67]. Our in vitro experiments suggested that Schisandrol A inhibit the NF-κB signaling pathway via inhibiting degradation of IκB by binding to β-TrCP (Figure 4D). Therefore, we found that Schisandrol A could prevent the progression of ar-thritis by inhibiting the NF-κB signaling pathway.

In conclusion, according to our experimental results, Schisandrol A inhibited the expression of MMP3, MMP13, and COX-2 factors known to cause cartilage destruction, by inhibiting the NF-κB signaling pathway (Figure 4D), and it suppressed cartilage destruction in a mouse model of degenerative arthritis (DMM model). Both in vitro and in vivo experiments provided evidence that the degradation of cartilage could be inhibited. Therefore, we propose that Schisandrol A is a potential candidate for the development of new drugs to treat OA.

## 4. Materials and Methods

### 4.1. Mice

In vivo animal experiments were approved by the Animal Care and Use Committee of the University of Ajou and complied with the Guide for the Care and Use of Laboratory Animals Eighth Edition published by the National Institutes of Health. All mice were purchased from DBL Co., Ltd. (Chungbuk, South Korea). C57BL/6J male mice weighing 18–20 g (10 weeks old) were housed at 23 °C and exposed to a 12/12 h light/dark cycle. Food and water were provided regularly. Five-day old Institute of Cancer Research mice were used for articular chondrocyte culture.

### 4.2. Culture of Articular Chondrocytes and Viability Analysis

Mouse articular chondrocytes were isolated from the femoral condyles and tibial plateaus of 5-day-old postnatal mice. Cartilage tissue was digested with 0.2% collagenase type II. The chondrocytes were seeded in 96-well dishes (9 × 103 cells/well) and incubated for 48 h prior to treatment. Schisandrol A was added at various concentrations (200, 400, 800, and 1000 μM), and the cultures were incubated in Dulbecco’s Modified Eagle Medi-um (DMEM) with 10% fetal calf serum (Capricon, Ebsdorfergrund, Germany), and 1% penicillin-streptomycin (Capricon, Ebsdorfergrund, Germany). After 24 h, we analyzed cell viability by assaying the culture medium for lactate dehydrogenase (LDH) activity using an LDH Colorimetric Assay Kit (BioVision, Inc., Milpitas, CA, USA). We used untreated samples (via-bility of 100%) and Triton X-100-treated samples (viability of 0%) for normalization. Viability was calculated using the following formula: 100-(sample LDH-negative control)/(maximum LDH-negative control) × 100. Each signal was measured with a SYNERGY H1 Microplate Reader (Biotek, Winooski, VT, USA) at 495 nm.

### 4.3. Reagents and Treatment

IL-1β was purchased from GenScript (Piscataway, NJ, USA). Schisandrol A and celecoxib were purchased from Sigma-Aldrich (St. Louis, MO, USA). Schisandrol A was dissolved in PBS at 50 mg/mL for oral administration to mice. Schisandrol A and celecoxib were dissolved in dimethyl sulfoxide (DMSO) for in vitro analyses, and IL-1β recombinant protein was dissolved in sterilized water. Mouse articular chondrocytes were treated with IL-1β (1 ng/mL) to create an in vitro OA environment and co-treated with Schisandrol A (400, 800, or 1000 μM) or celecoxib (50 μM, positive control) for 24 h before the cells were harvested.

### 4.4. Quantitative Reverse Transcription–Polymerase Chain Reaction (qRT-PCR)

Total RNA was isolated from articular chondrocytes using TRIzol (Molecular Re-search Center Inc., Cincinnati, OH, USA). The different primers used (e.g., for MMPs, GAPDH, and COX-2) are listed in Supplementary Table S1. The level of amplification of the target gene was evaluated by qRT-PCR using SYBR^®^ Green fluorescence and Premix Ex Taq (TaKaRa Bio, Kusatsu, Shiga, Japan). The transcription level of each target gene was normalized to that of GAPDH and expressed as fold-change relative to the indicated control.

### 4.5. Protein Isolation and Western Blotting

Whole protein was extracted from the primary cultured chondrocytes using RIPA lysis buffer containing 150 mM NaCl, 1% NP-40, 50 mM Tris/HCl (pH 8.0), 0.2% SDS, and 5 mM NaF with addition of protease and phosphatase inhibitor mixture (Roche, Madison, WI, USA). Total proteins were separated by SDS-PAGE, and Western blotting analysis was performed. The following antibodies were used: Goat anti-COX-2 (sc-1745; Santa Cruz, Dallas, TX, USA); mouse anti-ERK1/2 (610408; Becton Dickinson, NJ, USA); mouse anti-IκB (9242; Cell Signaling Technology, Danvers, MA, USA); mouse anti-p65 (8242; CST; mouse anti-phospho-p65 (3033; CST mouse anti-p38 (#9212; CST), mouse anti-pp38 (#9215S; CST); mouse anti-c-Jun N-terminal kinase (JNK) (#9252S; CST); mouse anti-pJNK (#9251S; CST); mouse anti-pErk (#9101S; CST). Each signal was visualized using the SuperSignal West Dura Kit (Thermo Scientific, Waltham, MA, USA). Density analysis (AlphaEase FC 4.0; Alpha Innotech, San Leandro, CA, USA) was used to quantify the relevant band intensities. Extracellular signal-regulated kinase (ERK) was used as the loading control.

### 4.6. PGE_2_, Collagenase, and Reporter Gene Assays

PGE_2_ production was evaluated using the PGE_2_ Immunoassay Kit (R&D System, Minneapolis, MN, USA). Primary mouse articular chondrocytes were seeded in 96-well plates (2 × 10^4^ cells/well). The levels of intracellular and secreted PGE_2_ were measured in total cell lysates. Total collagenase activity in the conditioned medium of the articular chondrocyte culture was determined using the EnzCheck Gelatinase/Collagenase Assay Kit (Molecular Probes, Carlsbad, CA, USA) and measured with a VICTOR X3 Microplate Reader (PerkinElmer, Waltham, MA, USA) at excitation/emission wavelengths of 490/530 nm, according to the manufacturer’s protocol. The NF-κB reporter gene plasmids were transfected into mouse articular chondrocytes via Lipofectamine Plus (Invitrogen, Carls-bad, CA, USA). After incubating the transfected cells for 24 h in complete medium, lucif-erase activity was assessed using an assay kit (Promega, Madison, WI, USA) and then normalized to β-galactosidase activity.

### 4.7. Experimental OA Mouse Model and Oral Administration

DMM surgery was performed on 10-week-old male C57BL/6J mice following a protocol used to create an OA model induced by medial meniscus disruption [62]. For histological analysis, the mouse knee joint was processed 10 weeks after surgery. In the DMM-induced OA model, Schisandrol A (5, 10, or 50 mg/kg) was administered daily by gavage for 6 weeks, and the mice were euthanized after completion of the 6-week regimen. There were four treatment groups (DMM + PBS, DMM + 5 mg/kg, DMM + 10 mg/kg, and DMM + 50 mg/kg). Three animals were used in each condition, and the total number of animals was 12.

### 4.8. Evaluation of Cartilage Destruction

Cartilage destruction was determined by Safranin O staining and scored using the OARSI grading system. Mouse knee joints were fixed with 4% paraformaldehyde, dehydrated with 0.5 M EDTA (pH 8.0) for 2 weeks, and embedded in paraffin. The paraffin blocks were successively cut at 50-μm intervals. The 50-μm pieces were then cut into 5-μm sections, which were fixed on glass slides. The sections were hydrated with a graded ethanol series, free of paraffin and xylene.

### 4.9. Immunohistochemistry

Paraffin sections were deparaffinized in xylene for immunohistochemical staining in cartilage tissue. Next, the sections were washed and, and hydrated with ethanol. In order to expose the Antigen of the sample, 0.1% trypsin was treated for 30 min. Sections were stained via the LSAB2 Horseradish Peroxidase Kit (Dako, Santa Clara, CA, USA) according to the manufacturer’s instructions. sections of the slide were incubated with an antibody against Adamts5 (ab41037, Abcam, Cambridge, UK) for 12 h at room temperature, followed by visualization of immunoreactive proteins using DACO AEC + high sensitivity matrix chromosome solution (Dako, Santa Clara, CA, USA).

### 4.10. Protein Structural Homology Modeling

Homology-based structural modeling of mouse β-TrCP (accession ID: NP_001032847) was performed using the SWISS-MODEL web server (http://swissmodel.expasy.org, accessed on 24 February 2021) [68]. The templates, human β-TrCP (PDB ID: 6TTU) was selected and the sequence similarities was 98.68% (QMEAN4 Z-scores given by SWISS-MODEL were −1.67). Chemical structure of Schisandrol A used in this study was retrieved from the PubChem database (https://pubchem.ncbi.nlm.nih.gov, accessed on, as of 24 February 2021) [69]. Molecular docking analyses were performed using AutoDock Vina (ver. 1.1.2) [70]. The receptor coordinates and the docking parameters have been prepared using AutoDock MGLTools (ver. 1.5.6) [71]. The graphical representation of docking structures was constructed using PyMOL (ver. 1.3; DeLanoScientific, San Carlos, CA, USA).

### 4.11. Statistical Analysis

The data were presented as means ± SEM. The entire histological sample was prepared independently by two researchers. Each experiment was performed at least three times. One-way ANOVA using the Bonferroni post-test was used for data analysis. For performing statistical analysis, we used PRISM 7 software and recognized significance as *p* ≤ 0.05.

## Data Availability

The data presented in this study are available in the main text and associated Supplementary Materials.

## Supplementary Materials

The following are available online at https://www.mdpi.com/1424-8247/14/3/241/s1, Supplementary Figure S1: β-TrCP (F-box/WD repeat-containing protein 1A) amino acid sequence. Blue: Shcisandrol A binding sites. Supplementary Table S1: PCR primers.
