# Supplementary material for: Schisandrol A Suppresses Catabolic Factor Expression by Blocking NF-κB Signaling in Osteoarthritis
Source: Pharmaceuticals (Basel). 2021 Mar 8;14(3):241. doi: 10.3390/ph14030241 (PMC7999623; doi:10.3390/ph14030241)
Supplement: Supplementary file 1 [file pharmaceuticals-14-00241-s001.pdf]

MDPAEAVLQEKALKFMCSMPRSLWLGCSSLADSMPSLRCLYNPGTGALTAFQN
SSEREDCNNGEPPRKIIPEKNSLRQTYNSCARLCINQETVCLTSTAMKTENCVAKA
KLANGTSSMIVPKQRKLSASYEKEKELCVKYFEQWSESDQVEFVEHLISQMCHYQ
HGHINSYLKPMLQRDFITALPARGLDHIAENILSYLDAKSLCAAELVCKEWYRVTS
DGMLWKKLIERMVRTDSLWRGLAERRGWGQYLFKNKPPDENAPPNSFYRALYP
KIIQDIETIESNWRCGRHSLQRIHCRSETSKGVYCLQYDDQKIVSGLRDNTIKIWDK
STLECKRILTGHTGSVLCLQYDERVIITGSSDSTVRVWDVNAGEMLNTLIHHCEAV
LHLRFNNGMMVTCSKDRSIAVWDMASPTDITLRRVLVGHRAAVNVVDFDDKYI
VSASGDRTIKVWNTSTCEFVRTLNGHKRGIACLQYRDRLVVSGSSDNTIRLWDIEC
GACLRVLEGHEELVRCIRFDNKRIVSGAYDGKIKVWDLMAALDPRAPAGTLCLRTL
VEHSGRVFRLQFDEFQIVSSSHDDTILIWDFLNDPAAHAEPPRSPSRTYTYISR

Supplementary Figure 1. β-TrCP (F-box/WD repeat-containing protein 1A) amino acid sequence. Blue : Shcisandrol A binding sites.

Gene	Origin	Strand	Sequence	Size (bp)	AT[a] (˚C)
Mmp3	Mouse	[b]S<br>[c]As	5'-CTGTGTGTGGTTGTGTGCTCATCCTAC-3'<br>5'-GGCAAATCCGGTGTATAATTCACAATC-3'	350	58
Mmp13	Mouse	S<br>As	5'-TGATGGACCTTCTGGTCTTCTGGC-3'<br>5'-CATCCACATGGTTGGGAAGTTCTG-3'	473	58
Cox-2	Mouse	S<br>As	5'-GGTCTGGTGCCTGGTCTGATGAT-3'<br>5'-GTCCTTTCAAGGAGAATGGTGC-3'	724	65
Adamts5	Mouse	S<br>As	5′-GCCATTGTAATAACCCTGCACC-3'<br>5′-TCAGTCCCATCCGTAACCTTTG-3′	280	58
Gapdh	Mouse	S<br>As	5'-TCACTGCCACCCAGAAGAC-3'<br>5'-TGTAGGCCATGAGGTCCAC-3'	450	55

[a]AT, annealing temperature; [b]S, sense; [c]As, antisense

Supplementary Table S1. Primer sequences and PCR conditions.
